# Dr. William Robinson

**Published:** 1905-11

**Authors:** 


Dr. William Robinson, of Chicago, died at his home October 10, 1905. He graduated from the University of Buffalo, medical department, in 1862.

